# Response inhibition in Attention deficit disorder and neurofibromatosis type 1 – clinically similar, neurophysiologically different

**DOI:** 10.1038/srep43929

**Published:** 2017-03-06

**Authors:** Annet Bluschke, Maja von der Hagen, Katharina Papenhagen, Veit Roessner, Christian Beste

**Affiliations:** 1 Cognitive Neurophysiology, Department of Child and Adolescent Psychiatry, Faculty of Medicine to the TU Dresden, Germany; 2 Abteilung Neuropädiatrie, Medizinische Fakultät Carl Gustav Carus, Technische Universität Dresden, Germany; 3 Experimental Neurobiology, National Institute of Mental Health, Czech Republic, Germany

## Abstract

There are large overlaps in cognitive deficits occurring in attention deficit disorder (ADD) and neurodevelopmental disorders like neurofibromatosis type 1 (NF1). This overlap is mostly based on clinical measures and not on in-depth analyses of neuronal mechanisms. However, the consideration of such neuronal underpinnings is crucial when aiming to integrate measures that can lead to a better understanding of the underlying mechanisms. Inhibitory control deficits, for example, are a hallmark in ADD, but it is unclear how far there are similar deficits in NF1. We thus compared adolescent ADD and NF1 patients to healthy controls in a Go/Nogo task using behavioural and neurophysiological measures. Clinical measures of ADD-symptoms were not different between ADD and NF1. Only patients with ADD showed increased Nogo errors and reductions in components reflecting response inhibition (i.e. Nogo-P3). Early perceptual processes (P1) were changed in ADD and NF1. Clinically, patients with ADD and NF1 thus show strong similarities. This is not the case in regard to underlying cognitive control processes. This shows that in-depth analyses of neurophysiological processes are needed to determine whether the overlap between ADD and NF1 is as strong as assumed and to develop appropriate treatment strategies.

Inhibitory control processes, required for the prevention of prepotent and inadequate responses, play an important role in everyday life^1^. Dysfunctions in these mechanisms represent a hallmark in attention deficit (hyperactivity) disorder (AD(H)D)^2^,^3^,^4^,^5^,^6^. However, ADHD symptoms are also found in other neurodevelopmental disorders, like neurofibromatosis type 1 (NF1)^7^,^8^,^9^,^10^,^11^. NF1 is a rare monogenetic, autosomal dominant genetic disorder caused by mutations in the tumor suppressor gene *neurofibromin* 1 (17q11.2, MIM*613113) in which a broad spectrum of cognitive deficits occur in 30–70% of cases. These mostly appear as learning deficits, attentional deficits, hyperactivity and language problems^7^,^8^,^11^,^12^,^13^, with full-scale AD(H)D being diagnosed in nearly every second child with NF1^14^. Of all symptoms, inattention is predominant in NF1^15^. Several lines of evidence from animal models suggest that NF1 is associated with dysfunctional dopaminergic neural transmission^16^,^17^,^18^,^19^, likely leading to deficits in attentional selection processes and hyperkinetic symptoms^16^,^17^,^20^. In such animal models, molecular links have also been demonstrated between ADHD-like locomotor behaviours, deficient dopaminergic transmission and neurofibromin 1^14^. Specifically, NF1 + /−GFAPCKO mice are characterised by reductions in the pre-synaptic dopamine transporter and behaviourally show reduced (non-)selective attention^16^,^17^. These neurobiological alterations show commonalities with ADHD, and attentional deficits in NF1 can successfully be treated using methylphenidate^9^,^21^. Recently, it has also been shown that NF1 is associated with response inhibition deficits^22^.

However, until now it is unclear how far response inhibition deficits and their neurophysiological mechanisms are comparable between patients with ADHD and those with NF1 and an accompanying ADHD symptomatology. Here, based on the pattern of symptomatology, a comparison of patients with NF1 and those with ADD (i.e. who are characterised by inattention but not by symptoms of hyperactivity/impulsivity) seems particularly useful^15^. Generally, current knowledge about similarities and differences between ADD and NF1 is mostly based on clinical measures of cognitive deficits^23^, but not on approaches allowing a fine-grained analysis of cognitive subprocesses e.g. combining experimental psychological and neurophysiological approaches. Yet, such an approach is of importance, as stressed by the research domain criteria (RDoC) initiative. RDoC is conceived as a dimensional system using different units of analysis (e.g. neurophysiology and behavior) that is independent from current disorder categories. Its goal is to generate classifications stemming from basic behavioural neuroscience, rather than starting with an illness definition and seeking its neurobiological underpinnings^24^. Concerning cognitive systems, the construct “cognitive control” and the subconstruct “inhibition” is central in neurodevelopmental disorders^25^ and is assumed to represent a relevant RDoC dimension^24^.

In the current study we therefore examine and compare response inhibition processes at the behavioural and neurophysiological level in ADD and NF1. This will provide insights into the nature of each of these disorders that have until now not been obtained. At the behavioural level, the rate of false alarms (i.e. responses in situations where the response has to be inhibited) is the most relevant parameter. At the neurophysiological level, different subprocesses from perceptual and attentional selection, to response selection and motor processes can be distinguished by examining event-related potentials (ERPs)^26^,^27^. Differences and similarities between ADD and NF1 may be based in one or several of these stages. Perceptual and attentional selection processes are reflected by the P1 and N1 ERPs^28^,^29^,^30^. Further along the processing cascade, mechanism related pre-motor processes like conflict monitoring or updating of the response program (reflected by the Nogo-N2) can be dissociated from evaluative processes of the successful outcome of inhibition (reflected by the parietal and central Nogo-P3)^26^,^31^,^32^,^33^,^34^,^35^,^36^. In the present study we compare adolescent patients with ADD and NF1 to healthy controls in each of the above processes to achieve a fine-grained picture of similarities and differences between these disorders in regard to response inhibition. This is crucial when aiming to integrate and synthesize measures which can lead to a better understanding of the mechanisms and in turn the symptoms to which they relate^24^. Subsequently, this could trigger the development of putative individualised therapeutic strategies.

## Results

### Behavioural data

The three groups differed regarding false alarms in Nogo trials (F(2,43) = 5.4; p = 0.008). Bonferroni post-hoc testing revealed that patients with ADD committed significantly more false alarms (51.8 ± 15.8%) in Nogo trials than healthy controls (31.8 ± 16.5%) (p = 0.003), indicating an inhibition deficit in the ADD group. Patients with ADD also committed more Nogo false alarms than those with NF1 (34.8 ± 19.8%) (p = 0.03). The difference between the patients with NF1 and the healthy controls was not significant (p = 0.64).

Furthermore, significant differences between the three groups (ADD: 92.9 ± 7.8%, NF1: 90.9 ± 10.3%; controls: 97.9 ± 2.7%) were found concerning the amount of correct responses in Go trials (F(2,43) = 3.9; p = 0.03). Bonferroni post hoc tests showed for the differences between the control group and the patients with NF1 to be significant (p = 0.01). This was not the case for any of the other comparisons (all p > 0.06). For the reaction times on Go trials the NF1 group (491 ± 135 ms) showed significantly slower response than patients with ADD (415 ± 55 ms, p = 0.02) and controls (416 ± 89 ms, p = 0.02) (F(2, 43) = 3.4; p = 0.042).

### Neurophysiological data

#### Perceptual categorization (P1) and attentional selection (N1)

**Figure 1:**
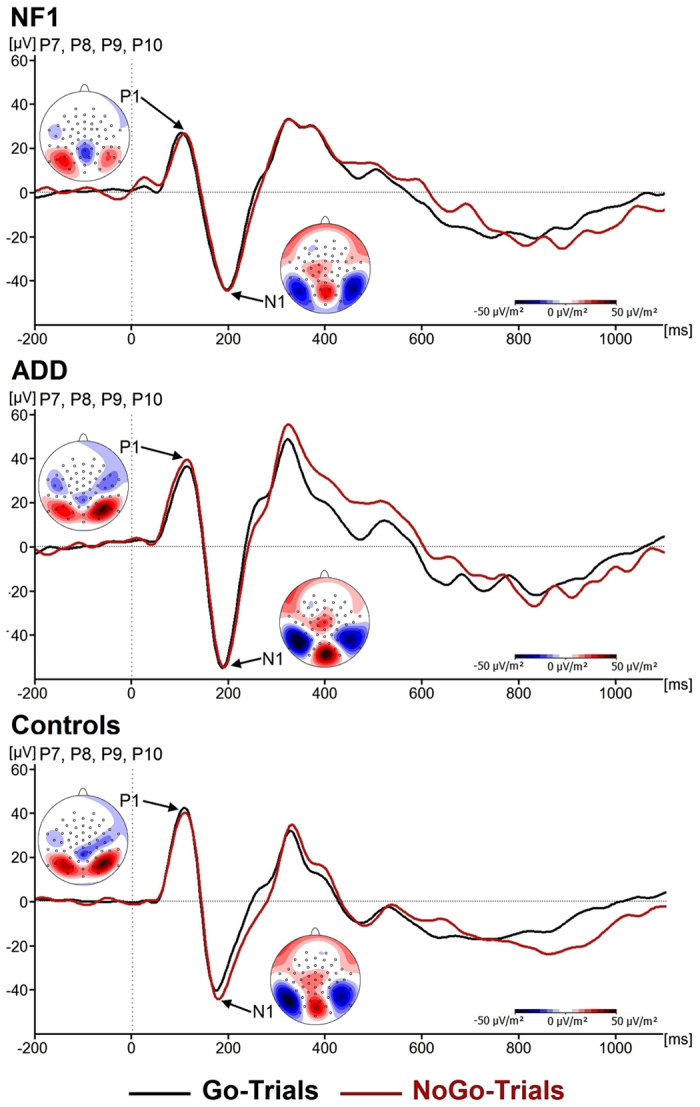
Event-related potentials (ERPs) showing the P1 and N1 component on Go and Nogo trials. The scalp topographies show the peak of the amplitudes. Positive values are given in red, negative values are given in blue. Time point zero denotes the time point of Nogo stimulus presentation. Negative values are plotted downwards. The NF1 patients are shown at the top, the ADD patients in the middle and the control at the bottom of the figure.

P1 and N1 components are shown in Fig. 1.

Regarding P1 amplitudes, analyses revealed a main effect of *Group* (F(2, 44) = 4.1; p = 0.02; η_p_^2^ = 0.16) as well as a trend level interaction of GoNogo*Group (F(2,44) = 2.9, p = 0.06, η_p_^2^ = .11). Further univariate analyses revealed significantly reduced P1 amplitudes in patients with NF1 (26.9 ± 5.9 μV/m^2^) compared to healthy controls (45.9 ± 5.1 μV/m^2^, p = 0.02) and patients with ADD (49.2 ± 5.4) μV/m^2^, p = 0.01). The difference between controls and patients with ADD was not significant (p = 0.66). Further analysis of the trend level interaction of GoNogo*Group revealed significant differences between Go (47.3 ± 4.5 μV/m^2^) and Nogo trials (42.3 ± 5.1 μV/m^2^) in healthy controls only (F(1, 16) = 5.3; p = 0.04, η_p_^2^ = 0.3). This difference was not significant in patients with ADD or NF1 (all F < 1.1; all p > 0.4). Concerning P1 latency, no main effects or interaction were significant (all F < 1.9; all p > 0.15; all η_p_^2^ < 0.08).

Concerning N1 amplitude, no significant main effects or interactions were found (all F < 2.8; all p > 0.1; all η_p_^2^ < 0.06). In terms of latency we found a main effect of *Group* (F(2,44) = 4.2; p = 0.02; η_p_^2^ = 0.16). Pair-wise comparisons showed that the N1 peak occurred significantly later in patients with NF1 (195 ± 15 ms) than in healthy controls (177 ± 16 ms) (p = 0.008). N1 latencies in patients with ADD (188 ± 15 ms) were not significantly different to those in patients with NF1 (p = 0.33) or healthy controls (p = 0.06).

#### Response selection processes (N2 as well as central and parietal P3)

**Figure 2:**
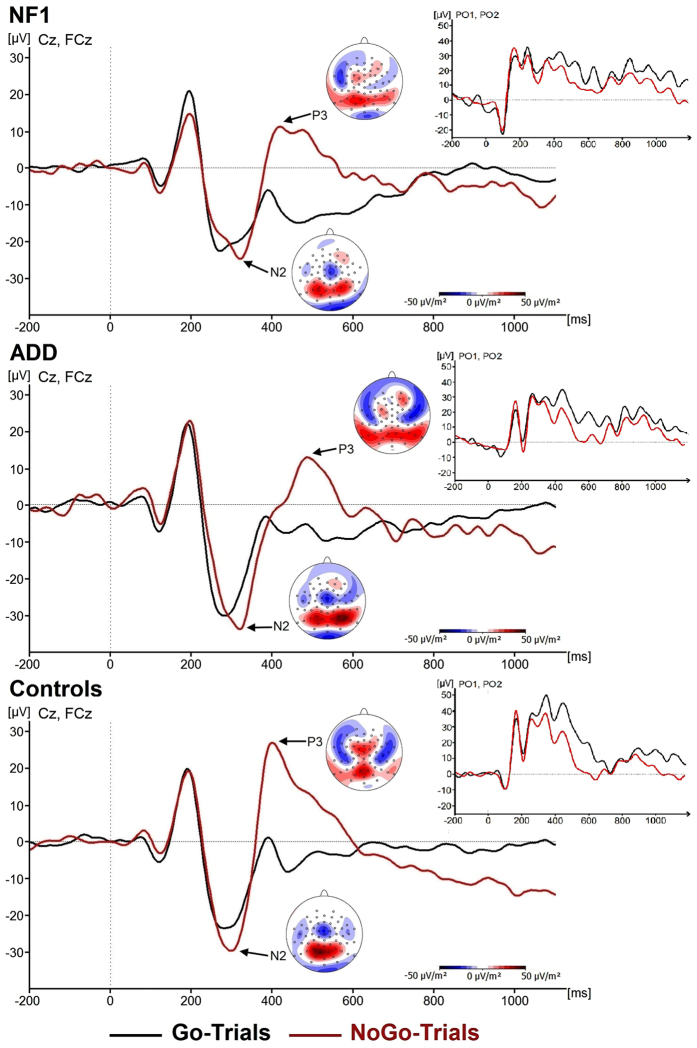
Event-related potentials (ERPs) showing the N2 and P3 component on Go and Nogo trials. The scalp topographies show refer to the peak of the amplitudes. Positive values are given in red, negative values are given in blue. Time point zero denotes the time point of Nogo stimulus presentation. Negative values are plotted downwards. The NF1 patients are shown at the top, the ADD patients in the middle and the control at the bottom of the figure. Inlays represent waveforms of the parietal P3 components over PO1 and PO2.

N2 and P3 components are shown in Fig. 2.

Analysing the N2 component, we found no main effects or interactions for either amplitude (all F < 0.2; all p > 0.66; all η_p_^2^ < 0.006) or latency (all F < 2.2; all p > 0.14; all η_p_^2^ < 0.05).

Concerning central P3 (cP3) amplitude, we found a main effect of *GoNogo* (F(1,44) = 8.4, p = 0.006, η_p_^2^ = 0.16), showing that the cP3peak was generally larger in Nogo (21.2 ± 21.6 μV/m^2^) than in Go (amplitude 0.16 ± 19.7 μV/m^2^) trials. Most importantly, there was an interaction of GoNogo*Group (F(2,44) = 10.9; p < 0.001; η_p_^2^ = 0.33). Examining this further, we found a main effect of *Group* in the Nogo trials (F(2,44) = 6.5; p = 0.004), but not in the Go trials (F(2,44) = 0.77; p = 0.47). Within the Nogo trials, patients with ADD (amplitude: 9.2 ± 19.2 μV/m^2^) had a significantly reduced cP3 peak compared to healthy controls (amplitude: 32.5 ± 25.7 μV/m^2^) (p = 0.001). The difference between patients with NF1 (amplitude: 21.9 ± 19.3 μV/m^2^) and those with ADD was also significant (p = 0.02), while this was not the case when comparing them to healthy controls (p = 0.55).

A similar pattern became apparent in regard to cP3 latency, where the interaction of GoNogo*Group was also significant (F(2, 44) = 4.1; p = 0.02; η_p_^2^ = 0.16). The main effect of *Group* was significant in the Nogo (F(2,44) = 4.2; p = 0.02) but not in the Go trials (F(2,44) = 1.7; p = 0.2). Within the Nogo trials, differences between patients with ADD (393 ± 32 ms) and healthy controls (408 ± 30 ms) were not significant (p = 0.3). However, the Nogo cP3 peak occurred significantly later in patients with NF1 (latency: 442 ± 49 ms) than was the case for patients with ADD (p = 0.007) and healthy controls (p = 0.05).

For the parietal P3 (pP3) (see Fig. 2), we only found a significant main effect of *GoNogo* (F(2, 44) = 7.7; p = 0.008; η_p_^2^ = 0.15), with pP3 peaks in Go trials (17.7 ± 3.9 μV/m^2^) being generally less pronounced than those in Nogo trials (21.9 ± 4.2 μV/m^2^). All other main effects and interactions were not significant (all F < 3.5; all p > 0.07; all η_p_^2^ = 0.08).

## Discussion

In the current study we examined behavioural and neurophysiological processes of response inhibition mechanisms in a sample of age-matched paediatric patients with ADD and NF1 as well as in healthy controls. The goal of this comparison was to evaluate how far ADD and NF1 show distinct and comparable behavioural and neurophysiological processes underlying response inhibition and impulsivity. This is important when aiming to integrate and synthesize measures which can lead to a better understanding of the mechanisms and in turn of the symptoms to which they relate^24^.

The clinical assessment of ADHD symptoms in both groups using the ADHD Symptom Checklist^37^ revealed that patients with NF1 and those with ADD are highly comparable. Thus, both patients groups fulfil criteria of ADD. The in-depth cognitive-neurophysiological assessment, however, revealed a more fine-grained picture. On the behavioural level, the results show that response inhibition processes are compromised in ADD, but not in NF1 when compared to healthy controls. The finding that there was no difference between controls and patients with NF1 stands in contrast to other recent results on response inhibition processes in NF1^22^. There are explanations for this. Firstly, in the study by Riberio *et al*.^22^ the level of possible ADD symptoms was not assessed and the age range of patients was broader. Another explanation for the difference in behavioural findings is that the paradigm used by Riberio *et al*.^22^ was more difficult, because only one out of nine stimuli was a Nogo stimulus and all stimuli were presented at equal frequency. This procedure minimized the possibility that attentional templates could support task performance. The Nogo task in the present study was easier to perform. It is therefore likely that response inhibition deficits in NF1 are less strong compared to those occurring in ADD. This result is supported by the neurophysiological data. Here, healthy controls showed the largest Nogo-cP3 amplitudes. However, only patients with ADD differed from controls. This was not the case for the patients with NF1. The difference between the two patient groups was significant and cP3 amplitudes in patients with NF1 are thus not deficient as in patients with ADD. These differential effects were driven by neurophysiological processes during Nogo trials, i.e. mechanisms related to response inhibition. Regarding the N2, no differences between groups were evident. This shows that differences between ADD and NF1 are highly specific and affect motor processes of response inhibition, but not premotor inhibitory control processes reflected by the Nogo-N2. Several results demonstrate that it is specifically the Nogo-P3 which is altered in ADD and ADHD^2^,^3^,^38^,^39^,^40^,^41^. Interestingly, it has been shown that processes reflected by the Nogo-N2 and Nogo-P3 depend on dissociable dopaminergic systems; i.e. the nigrostriatal and dopamine D1 receptor system in case of the Nogo-N2, and the mesocortico-limbic and dopamine D2 receptor system in case of the Nogo-P3^32^,^42^. Especially reduced dopamine D2 turnover is associated with a reduced motor inhibition processes and a reduced Nogo-P3 amplitude^42^. The cognitive-neurophysiological results therefore suggest that especially the meso-corticolimbic and/or dopamine D2 receptor system may show differences between ADD and NF1. Such possible differences in the catecholaminergic system between ADD and NF1, which certainly need to be examined in more detail, may also influence the way in which effects of methylphenidate on ADD-symptoms in children with NF1 are examined in the future. Although results so far have been promising^9^,^21^, the knowledge of such possible differential underlying mechanisms may be helpful for the optimisation and individualisation of treatment approaches.

Despite these differences between NF1 and ADD at the level of cognitive control, mechanisms of attentional selection (cf. N1 ERPs)^28^ were not differentially modulated between these diseases and were also not different to controls. Interestingly, perceptual categorization processes, reflected by the P1^43^ were altered in NF1 and ADD. Firstly, P1 amplitudes were generally lower in patients with NF1 compared to the other two groups. Secondly, controls clearly show distinct responses between Go and Nogo stimuli. This distinction, however, seemed to be almost abolished in patients with ADD and NF1. This perceptual deficit may contribute to the response inhibition deficit in ADD. Yet, it does not seem to do so in NF1, because response inhibition performance was not different to controls. However, the generally reduced early perceptual processing, which was exclusively observed in patients with NF1, could be related to the reduced accuracy in Go trials, which also only occurred in this group. This reduced accuracy can, of course, also be explained by the general motor slowing (cf. reaction times) seen in NF1 compared to ADD and controls. In response inhibition tasks, it is the speeding of response that emerges due to the frequency of Go trials (responses) that makes it hard to inhibit this response^44^,^45^,^46^,^47^. What therefore counts for the performance in response inhibition are speeded reactions on Go trials making it hard to inhibit the prepotent responses on Nogo trials. Due to the relative slowing of RTs in patients with NF1, response inhibition may thus be less demanding. There is a speed-accuracy trade-off in NF1 patients not seen in ADD patients, which may explain why response inhibition in NF1 is not as dysfunctional as in ADD. An important clinical implication of this finding is that therapeutic attempts to increase motor performance in NF1 may bear the risk that important executive control functions are affected negatively. It also needs to be considered how the observed similarities and differences between patients with NF1 and those with ADD would change across development from childhood to adulthood. Partly based on the maturation of the dopaminergic system during adolescence, only around 15% of children with ADHD are estimated to also meet full diagnostic criteria at the age of 25^48^. Based on its aetiology, NF1 is a lifelong disorder and neurocognitive deficits have been shown to persist into adulthood^49^. Thus, the magnitude of the differences between ADD and NF1 observed in the current study may even increase in adulthood, although specific trajectories remain to be investigated and compared between the two groups as well as health controls.

Even though the obtained results are robust, a clear limitation of the study is the limited sample size. The study’s results will therefore require replication. Moreover, only one instance of cognitive control processes was examined. Other dimensions, e.g. those related to interference control and different aspects of attentional selection, deserve further detailed investigations. In addition it is important to consider that some of the patients with ADD did receive stimulant medication. Sample sizes were not sufficient to differentially consider the effects of medication on performance, but previous research^50^ suggests that P3 amplitudes would potentially have been even lower if only medication-naïve patients with ADD had been included in the current study. Nevertheless, this is a very important aspect to consider in future investigations. Especially, it would be interesting to examine the specific effects of stimulant medication on the neurophysiological correlates of response inhibition in children with NF1 and their relation to reported symptom improvements in this patients group^9^,^21^.

To summarize, according to standard clinical measures assessing ADD symptoms, both diseases show a considerable overlap. However, an in-depth analysis of neurophysiological subprocesses involved in response inhibition as an important instance of cognitive control processes provides a more differentiated picture. The results show that patients with ADD and NF1 both display dysfunctions in perceptual categorization and that this process is additionally generally significantly attenuated in patients with NF1. Yet, aside from this dimension of cognitive processes, patients with ADD show response inhibition deficits (compared to controls) which are not seen in NF1, despite “impulsivity” being comparable when considering clinical measures. The results suggest that clinical measures of symptoms should be complemented by cognitive-neurophysiological approaches. These are necessary when aiming to determine whether the overlap between ADD and NF1 is as strong as currently assumed and to gain a better understanding of the mechanisms and symptoms to which they relate to inform individualized treatment strategies.

## Materials and Methods

### Samples

All subjects and their parents or legal guardians provided informed written consent according to the Declaration of Helsinki and the study was approved by the local ethics committee of the Medical Faculty of the TU Dresden.

N = 13 patients with NF1 were included in the study (9 female, 3 brothers, age 13.5 ± 2.5 years). The NF1 diagnoses were based on the clinical criteria by the National Institutes of Health (NIH) Consensus Development Conference on Neurofibromatosis (NIH, 1988).

N = 16 adolescent patients diagnosed with ADD (1 female, 12.9 ± 3.1 years) according to ICD-10 criteria were recruited consecutively into the study from the outpatient clinic. 6 of these patients were taking medication (immediate or extended release methylphenidate or atomoxetine). Standard clinical procedures (incl. parent and child interview, teacher report, symptom questionnaires, IQ testing, exclusion of potential underlying somatic disorders via EEG, EKG, audiometry and vision testing) were used to confirm the diagnosis of ADD. Children were only included in the study if they fulfilled diagnostic criteria for ADD.

N = 17 children without ADHD or NF1 were included in the control group (6 female, 13.9 ± 3.4 years). None of them were taking medication and none had a psychiatric diagnosis as confirmed by clinical interview. Using the ADHD Symptom Checklist^37^ parents rated (0: no problems, 3: severe problems) their children in regard to inattention, hyperactivity and impulsivity. Based on this questionnaire, both patient groups fulfilled criteria of ADD according to the ICD-10 criteria (F98.8). Patients with NF1 (1.4 ± 0.8) did not differ from those with a confirmed diagnosis of ADD (1.9 ± 0.5) in regard to inattention (p = 0.13). In the hyperactivity dimension (ADD: 0.7 ± 0.5; NF1: 0.4 ± 0.6), the impulsivity dimension (ADD: 1.2 ± 0.7; NF1: 1.0 ± 0.9) and in the overall score (ADD: 1.2 ± 0.3; NF1: 1.0 ± 0.7), the groups did not differ from each other (all p > 0.48). Age did not differ significantly between the groups (p > 0.13). Gender distribution was significantly different between the groups (χ^2^(2) = 7.2; p = 0.03). To account for this difference, *Gender* was included as a covariate in all analyses. The authors assert that all procedures contributing to this work have been conducted in accordance with the ethical standards of the relevant national and institutional committees on human experimentation and with the Helsinki Declaration of 1975, as revised in 2008. The study was approved by the local ethics committee of the Medical Faculty of the TU Dresden.

### Task

We used a standard Go/Nogo task^51^, which was also used previously to examine response inhibition deficits in ADHD^3^,^52^ during the task one out of two words was presented on a monitor: ‘DRÜCK’ (German for ‘PRESS’; Go stimulus) and ‘STOP’ (German for ‘STOP’; Nogo stimulus) were presented for 300 ms. Participants were asked to respond fast (i.e. within 500 ms) on the ‘DRÜCK’ stimulus and refrain from responding on the ‘STOP’ stimulus. The subjects had to react with the right index finger. The inter-trial interval (ITI) was jittered between 1600 ms and 1800 ms. The experiment consisted of 248 Go trials and 112 Nogo trials presented in a pseudo-randomized order to avoid consecutive identical trial conditions. The task lasted approximately 20 minutes.

### EEG recording and analyses

The EEG was recorded from 60 Ag/AgCl electrodes (500 Hz sampling rate). Electrode impedances were kept below 5 kΩ. The reference electrode was located at Fpz and the ground electrode was located at θ = 58, ф = 78. During off-line data processing, the data were down-sampled to 256 Hz. Afterwards, a band-pass filter from 0.5 to 20 Hz (slope of 48 db/oct) was applied. After removing technical artifacts, periodically occurring artifacts (pulse artifacts, horizontal and vertical eye movements) were detected and corrected for by means of an independent component analysis. Then, the EEG was segmented to the onset of the Go and Nogo stimuli. Only trials with correct responses on Go and without responses on Nogo trials were considered. Segments started 200 ms before and ended 1500 ms after stimulus onset. An automated artefact rejection procedure was applied containing an amplitude criterion (maximal amplitude: 200 μV, minimal amplitude: −200 μV) and using a maximal value difference of 200 μV in a 200 ms interval as well as an activity below 0.5 μV in a 100 ms period as rejection criteria. Next, a current source density (CSD) transformation was run to obtain a reference-free evaluation of the EEG data which helps to find the electrodes showing the strongest effects^53^. A baseline correction was then set to a time interval from −200 ms to 0 ms before the segments were averaged for each condition. For ERP quantification the following electrodes were chosen on the basis of the scalp topography. Single-subject ERP-amplitudes were quantified semi-automatically as the most positive/negative peak (amplitude and latency) within a defined time interval: The P1 peak was measured over pooled electrodes P7, P8, P9 and P10 in the time window of 90–120 ms post-stimulus. The N1 peak was quantified over the same electrodes in the time window of 165–205 ms after the stimulus. Electrodes FCz and Cz were pooled and then used to measure the N2 (250–330 ms) and central P3 peaks (cP3, 380–430 ms). To account for parietal P3 components (pP3) which especially occur in Go trials and shift towards more anterior regions in Nogo trials (central P3)^26^,^54^, we also examined activation at electrodes PO1 and PO2 in the time window of 330–350 ms. As no clear peak was discernible in the visual inspection of the data in case of the pP3, an area export was performed instead of a peak detection. This choice of electrodes and time windows was validated using a statistical procedure described in ref. 55.

### Statistics

Behavioural data were analyzed using univariate ANOVAs and t-tests. The neurophysiological data were analyzed by means of mixed effects ANOVAs using the within-subject factor *Condition* (Go vs. Nogo) and the between-subjects factor *Group* (NF1 vs. ADD vs. controls). P1 and N1 components were analysed over electrodes P7, P8, P9 and P10 (pooled together). P2, N2 and cP3 were quantified at electrodes Cz and FCz (pooled together). pP3 was examined at electrodes PO1 and PO2 (pooled together). Greenhouse-Geisser correction was applied and post-hoc tests were bonferroni-corrected when necessary. To account for differences in gender distribution between the three groups, Gender was included as a covariate in all analyses. All variables were normally distributed as indicated by Kolmogorov-Smirnov tests (all z < 1.05; p > 0.2).

### Ethical standards

The authors assert that all procedures contributing to this work have been conducted in accordance with the ethical standards of the relevant national and institutional committees on human experimentation and with the Helsinki Declaration of 1975, as revised in 2008. The study was approved by the local ethics committee of the Medical Faculty of the TU Dresden.

## Additional Information

**How to cite this article:** Bluschke, A. *et al*. Response inhibition in Attention deficit disorder and neurofibromatosis type 1 – clinically similar, neurophysiologically different. *Sci. Rep.* **7**, 43929; doi: 10.1038/srep43929 (2017).

**Publisher's note:** Springer Nature remains neutral with regard to jurisdictional claims in published maps and institutional affiliations.
